# Women’s experiences of high-risk pregnancy care in resource constrained Cape Town communities

**DOI:** 10.4102/hsag.v30i0.2890

**Published:** 2025-05-27

**Authors:** Gugulethu Cebekhulu, Michelle G. Andipatin

**Affiliations:** 1 Department of Psychology, Faculty of Community and Health Sciences, University of the Western Cape, Cape Town, South Africa

**Keywords:** high-risk pregnancy, qualitative, government healthcare services, quality of care, subjective experiences, resource-constrained, Western Cape, South Africa

## Abstract

**Background:**

The ‘high-risk’ classification during pregnancy leads to constant monitoring and frequent interactions with healthcare professionals, making it crucial for healthcare providers to show compassion.

**Aim:**

The study aimed to describe how women diagnosed with a high-risk pregnancy experienced their pregnancies as well as their interactions with the government healthcare system in Cape Town.

**Setting:**

The study was carried out using the Zoom digital platform and telephone. Participants lived in Cape Town neighbourhoods that are traditionally referred to as townships.

**Methods:**

A qualitative exploratory research design was used in the study. Nine women over 18 years old, diagnosed as having had a high-risk pregnancy and had given birth within 2 years were purposively selected. Open-ended questions were utilised, and data were interpreted using a thematic analysis.

**Results:**

Four main themes with 10 sub-themes emerged. The main themes included: ‘Being labelled as high-risk’, ‘locus of control’, ‘fear’ and ‘hospitalisation’.

**Conclusion:**

The study revealed that the psychological requirements of women diagnosed with high-risk pregnancy are not always met by the healthcare system. Fear experienced by women emerged from the high-risk label itself, and the amplification of the risk status by clinicians.

**Contribution:**

Through the lens of expectant mothers utilising government healthcare services in Cape Town, the study gives insight into pregnant women’s experiences. This insight provides opportunities for healthcare providers to re-consider and incorporate some interventions that could assist women.

## Introduction

A high-risk pregnancy elicits inherent fear for women, which has an impact that is great and far-reaching, often resulting in psychological health challenges both during and after pregnancy (Minnaar 2020). Pregnancy is classified as high-risk when there is a possibility of difficulties during pregnancy, birth or the postpartum period, for either the mother or the growing baby (Soh & Nelson-Piercy 2015). In low- and middle-income countries (LMICs), where 99% of all maternal fatalities take place, women are more vulnerable to experiencing severe morbidity and mortality during pregnancy, delivery and the postpartum period (Heitkamp et al. 2021).

It is widely known that healthcare institutions in LMICs generally face considerable limitations (Meghji et al. 2021). Therefore, socioeconomic factors that are highly linked to morbidity and mortality have had a significant influence on the health of the majority of South Africans (Militao et al. 2022).

Individuals in impoverished regions face systemic limitations that influence their behaviour (Vilar-Compte et al. 2021). For example, they are more likely to consume unhealthy food because of the limited access to nutritious grocery shopping options beyond convenience stores, liquor stores and fast-food establishments, which primarily serve high-fat, high-sugar and high-salt items (Vilar-Compte et al. 2021). These unhealthy alternatives may often lead to many women being at risk for major health issues, including obesity, hypertension and gestational diabetes, which might endanger both their lives and the lives of their unborn children (Langley-Evans 2022). Consequently, despite the South African government’s efforts to enhance care for expectant mothers and children, disparities in perinatal and maternal outcomes predominate and continue (Ngene, Khaliq & Moodley 2023).

Although South Africa’s health system was formerly segregated based on race, it continues to reflect the social divisions of the nation (Maphumulo & Bhengu 2019). For example, there are currently two healthcare systems – the public healthcare system which serves the poor majority and the private healthcare system which caters to those who occupy the higher socioeconomic status. Despite South African citizens being able to access free healthcare through the public healthcare system, a major drawback is that it is grossly underfunded despite servicing a large percentage (84%) of the population (Maphumulo & Bhengu 2019; Mhlanga & Garidzirai 2020). This challenge often manifests as long waiting periods for patients and a highly stressful working environment for healthcare professionals (Crush & Tawodzera 2014; Wium, Vannevel & Bothma 2019).

In addition, South Africa’s healthcare system is underpinned by the biomedical model, which views the human body as a mechanical, universal object that is devoid of culture (Pentecost et al. 2018). According to this line of thinking, pregnancy is viewed as purely physical, which undercuts the significance that culture and experience have on pregnant women’s interpretation of their experiences (Nuño de la Rosa, Pavličev & Etxeberria 2021). Thus, interactions with clinical professionals often do not provide women with the necessary emotional and psychological fulfilment that they require.

Furthermore, African traditions and beliefs, which promote wholeness, a treatment approach that considers the full individual, including their social environment, stand in sharp contrast to the biomedical model (Holst 2020). As a result, this system challenges South African society’s conventional, moral, and spiritual values. Moreover, according to the biological model paradigm, a pregnant woman is automatically at health risk, categorised as either ‘high–risk’ or ‘low–risk’ (Majella et al. 2019). As a result, pregnancy has taken on a techno-medical aspect as medical experts approached it like a disease rather than something that is normal and natural (Majella et al. 2019). Pregnant women are classified as ‘high-risk’ based on statistics rather than personal factors (Van Teijlingen et al. 2005).

This has given rise to the claim that giving birth is only safe in a hospital with medical staff present (Yuill et al. 2020). Thus, a ‘high-risk’ label subsequently leads to intense monitoring and frequent interaction with healthcare providers (Heemelaar et al. 2020). The ‘high-risk’ classification is an extremely stressful event that is intrinsically terrifying for women (Minnaar 2020), making it crucial for healthcare providers to show compassion. However, the biomedical framework uses technical language which often leaves patients feeling left out as they are unable to comprehend their own diagnosis (Khan 2019).

There are very few studies that have looked at women’s subjective experiences after a high-risk pregnancy in the setting of South Africa. Despite it being well recognised that pregnancy affects a woman’s overall state of health and emotional well-being (Abrar et al. 2020; Cole-Lewis et al. 2014), pregnancy is still viewed as a techno-medical event as researchers persist in concentrating on the physical conditions, ignoring the psychological distress that mothers face (Nagar et al. 2015; Torabi et al. 2012).

Women from developing countries are more exposed to stress and risk factors such as socioeconomic deprivation and poverty, increasing the likelihood for developing mental health problems (Nielsen-Scott et al. 2022). Given that one in five women residing in LMICs experience a mental disorder during and after pregnancy (Mitchell et al. 2023), it is unclear how South African women diagnosed with high-risk pregnancies interact with the healthcare system. Thus, the purpose of this study is to address this gap by investigating how a sample of women from resource-deprived neighbourhoods diagnosed with a high-risk pregnancy experienced the South African public healthcare system.

## Research methods and designs

### Research design

The study utilised a qualitative research design to understand the experiences of mothers who were diagnosed with a high-risk pregnancy. This design is useful for gaining a deeper understanding of life experiences in relation to social and cultural factors (Muzari, Shava & Shonhiwa 2022).

### Research setting and context

Participants of the study lived in Gugulethu, Mandalay, Mitchells Plain, Philippi and Nyanga, which are neighbourhoods that are traditionally known as townships in Cape Town, South Africa. Townships are often characterised by unfavourable living conditions, including extreme poverty, high levels of unemployment rates and a lack of reasonable infrastructure (adequate educational facilities, health, housing, recreational and transport facilities) (Donaldson et al. 2023). There is a high-birth rate, with most women having three to four children (Christodoulou et al. 2022; Lefulebe, Van der Walt & Xulu 2023). In many cases, women live within 5 km of a prenatal clinic, with various NPOs also within reach (Christodoulou et al. 2022).

### Sampling

Purposive sampling, a non-probability sampling technique was utilised for the study. This sampling technique allows the researcher to intentionally sample a group of people that will best inform the research problem of the study being conducted (Thomas 2022). The technique does not focus on the number of participants in the study, but is rather concerned with obtaining a comprehensive understanding of the research questions (Thomas 2022). Women were purposely selected if they were above the age of 18, had given birth in a Cape Town healthcare government facility and had recieved a high-risk diagnosis during pregnancy. Those who did not meet this criteria were excluded. Out of 12 participants who showed interest in the study, 3 did not meet the criteria.

### Data collection

Data was collected between September and October 2021. The first author conducted the data collection by interviewing participants in English and iSiXhosa. Interviews were conducted both telephonically and on the Zoom digital platform, as these were the two methods preferred by the participants. The duration of the interviews was between 22 min and 42 min. Prior to each interview, an information sheet was sent to the participants via WhatsApp. This was followed by a WhatsApp message to schedule a telephone call. This initial call aimed to establish rapport with participants, to provide a detailed explanation of the study and, at the same time, scheduling a suitable time for the interview.

Between interviews, the researcher held debriefing meetings with the supervisor. Follow-up interviews were conducted for participants to assess whether a referral for psychological support was necessary.

The study aimed to explore how women experienced the healthcare system during their high-risk pregnancy. This was achieved by asking clarifying and probing questions, such as:

- *Can you please tell me about your high-risk pregnancy?*
- *Which feelings dominated your experience while going through the pregnancy?*
- *How did you experience the birthing and birth of your baby?*

After interviewing nine participants, no new data emerged and therefore the number was deemed sufficient as saturation was reached.

### Data analysis

A thematic analysis was employed to analyse the data, and the analysis was carried out in accordance with the guidelines supplied by Percy, Kostere and Kostere (2015). The authors familiarised with and immersed themselves in each participant’s data. Following this, each piece of data was coded while making use of the research question as a guide. This process involved searching, identifying and categorising distinct concepts and themes (Williams & Moser 2019). Unrelated data to the research question were stored separately for potential later use. Next, a pattern was formed by grouping together all related items. Thematically similar patterns were grouped using direct excerpts from the transcribed interviews. The meanings of patterns that had nothing to do with previously established themes were kept separate and subsequently examined to determine whether they had any bearing on the subject as a whole.

To identify any emergence of overarching themes, each pattern was examined. Following the analysis of all the data, themes were grouped according to the supporting patterns. Patterns that did not fit into the pre-established categories were then re-examined. The researcher looked for any new themes and patterns that may have resulted from the data analysis and were relevant to the research question. Each theme underwent a detailed analysis. Finally, each theme was summarised along with a corresponding quote that linked it to the study’s goal and research question. Interviews were transcribed and coded by the first author, with co-coding to improve reliability and validity of the findings by the second author.

### Trustworthiness

Considerations such as credibility, transferability, dependability and confirmability must be addressed in order to quarantee legitimacy in a qualitative research study. In the present study, a semi-structured interview guide was used to enhance credibility.

Credibility is the degree of accuracy in the interpretation of the findings of a research study (Ahmed 2024). In a qualitative study, credibility is demonstrated by the researcher through extended engagement, methods of observation, triangulation, member checking, peer debriefing, referential adequacy, negative case analysis and by providing an audit trail (Ahmed 2024). To ensure credibility in this study, the researcher strived to ensure that the responses were accurately interpreted. In addition, careful attention was paid to the participants’ emotions during the interview process. Participants were allowed to confirm whether the researcher had appropriately interpreted their responses in the data form through member checking.

Dependability pertains to the study’s consistency across time, researchers and analytical methods (Ahmed 2024). In this study, dependability has been ensured by providing specifics of the data-gathering process. In addition, a peer debriefing method has been utilised, whereby two peers, fellow Masters research students, evaluated whether the findings, interpretations and conclusions corroborated the data. Furthermore, the researcher collaborated closely with the supervisor to investigate various aspects of the research study, including biases, understandings, interpretations, methodological considerations and ethical processes. During the analysis and final report writing, the supervisor also critically reviewed several conclusions and provided fresh perspectives.

Confirmability is the process of ensuring as far as possible that research findings are based on the experiences and perspectives of the researcher rather than references of the researcher (Ahmed 2024). To demonstrate confirmability in this study, the investigator has recognised any underlying views, decisions and techniques used in the research report. The study’s research design section provides an explanation of the rationale for the preference for a qualitative exploratory technique.

Transferability is the extent to which findings can be applied to other settings (Ahmed 2024). This was achieved by extensively detailing the methods and context.

### Ethical considerations

Ethical clearance to conduct this study was obtained from the University of the Western Cape, Biomedical Science Research Ethics Committee (ethics reference no.: BM21/5/16). All ethical principles and protocols were strictly adhered to. Participants were made aware that they could withdraw from the study at any time without facing any consequences. All participants were made aware of free counselling services such as the South African Depression and Anxiety Group (SADAG), LifeLine and other counselling services, should they require any support. Following the interviews, participants received a debriefing and follow-up calls to ensure that they were still doing well. To maintain privacy, the author conducted interviews in a private office. All recordings were kept confidential and the data were securely stored on a password-protected laptop. Consent was obtained verbally and recorded as part of the interview process. Confidentiality was maintained by allocating numbers to participants and not using their real names.

## Results

Table 1 presents the demographic characteristics of the sample, categorised by age, language, relationship status, place of residency, time frame of the high-risk pregnancy and the name of the high-risk diagnosis.

**TABLE 1:** Sample demographics.

Participant	Age (years)	Language	Relationship status	Place of residency	Last high-risk pregnancy	High-risk diagnosis
1	40	isiXhosa	In a relationship	Mandalay	24 months ago	Hypertension
2	38	isiXhosa	In a relationship	Philippi	18 months ago	Appendicitis
3	40	isiZulu	In a relationship	Gugulethu	1 week ago	Hypertension, diabetes
4	34	isiXhosa	Married	Gugulethu	24 months ago	Hypertension, preeclampsia, diabetes
5	32	English	In a relationship	Mitchells Plain	20 months ago	Hypertension, Preeclampsia
6	30	Afrikaans	Married	Mitchells Plain	4 months ago	Polycystic ovarian syndrome (PCOS)
7	40	isiXhosa	Single	Nyanga	23 months	Preeclampsia
8	32	isiXhosa	Married	Mandalay	6 months ago	Hypertension
9	35	isiXhosa	Married	Nyanga	24 months ago	Anaemia

### Theme 1: Being labelled as ‘high-risk’

Being labelled as ‘high- risk’ refers to the clinician’s projection of the likely adverse result of a pregnancy. This is based on a general risk profile rather than participants’ individual circumstances. Being classified as ‘high-risk’ was a key subject in the study, which affected how participants perceived and ultimately experienced their pregnancies. During their initial consultations with clinicians, several participants were advised of the potential poor outcomes, which were solely based on statistical projections. For instance, as can be seen in the excerpts below, Participants 1 and 3 were classified as ‘high -risk’ based solely on their age:

‘When I went to book at the clinic, I was sent to the hospital. They said that I was at high-risk because of my age, because at that time, I was 39.’ (Participant 1)

[*T*]they said because of my age, because I’m 40, it’s possible for me to have a miscarriage, if not the baby won’t be normal. So, they gave me a study about that 100 out of 500, something like that, mothers at my age …’ (Participant 3)

After experiencing unusual symptoms in her second trimester, Participant 5 was examined and diagnosed with hypertension and preeclampsia. She was subsequently advised that it was doubtful that she would be able to have any more children after receiving this diagnosis. Given that this was the young mother’s first pregnancy, hearing this was obviously distressing for her:

‘[*T*]he doctor told me that because of my first experience I might not be able to have children again, because I’ll always get sick. So, it’s very traumatising and heart breaking.’ (Participant 5)

### Theme 2: Locus of control

‘Locus of control’ refers to ‘the degree to which individuals believe their lives are controlled by themselves or external forces’ (Eswi & Khalil 2012:463). The sub-themes under the main theme included ‘mothers taking full control of birth outcomes’, ‘defying biomedical rules’ and ‘loss of control to clinicians’.

#### Sub-theme 2.1: Mothers taking full control of birth outcomes: Defying biomedical rules

The above-mentioned theme refers to participants who fully asserted control over the course of their pregnancies by rejecting treatments and procedures that they deemed potentially harmful to themselves and their unborn children. These individuals took control of the situation, disregarded the data provided to them on the hazards and instead trusted their own gut feelings.

For example, Participant 2’s appendicitis was diagnosed in the seventh month of her pregnancy. The medical staff warned her that carrying the baby could endanger her life and that, in such a case, the best course of action would be to save the mother’s life first. However, the participant objected to performing a C-section before full term, despite it being necessary to achieve success in this case. Below is her narrative:

‘So, they performed the operation but also told me about the chances. But I also told them that you used to doing things a certain way to people, but my baby will be fine. Then they said that they would keep me for observation since they didn’t do things the way that they normally do it, but I kept saying to them, that no, I will give birth at 9 months.’ (Participant 2)

Participants also considered the impact of suggested treatments and decided what they thought was best for their situations:

‘The test was to check if the baby had any disability, so what they told me was the test is risky, it can let you have a miscarriage or it may cause some kind of damage to the baby. So I said no … if my baby is not ok, then I’ll live with that when the time comes.’ (Participant 3)

Even though participants were reluctant to comply because of the perceived risk of the medical intervention to their unborn babies, this did not make the choice any easier. As shown here, it caused Participant 3 a great deal of tension and distress:

‘I had mixed, mixed feelings which resulted to having a very low BP and the doctor said that it was anxiety, I can remember.’ (Participant 3)

‘They wanted to admit me now to induce labour, and I refused, I said no! I want a second opinion. I don’t believe you guys and whatever.’ (Participant 5)

These actions do not necessarily indicate the participants’ lack of understanding of risks involved in their high-risk pregnancies; rather, they suggest that they were not satisfied with the clinicians’ decisions to intervene in their pregnancies.

#### Sub-theme 2.2: Loss of control: Releasing control to clinicians

While some participants felt completely in charge of their pregnancies, others felt compelled to give doctors that power, particularly, when unanticipated events occurred during their pregnancies. Hence, the theme ‘releasing control to clinicians’ describes participants who perceived their circumstances to be beyond their control. These participants described situations of having ‘no power and no control’ in their birthing process and pregnancy outcomes, especially when their pregnancies took an unpredictable turn for the worse.

For example, one participant’s pregnancy suddenly shifted to high-risk during labour. She was astonished and unprepared, especially because she had complied with all the suggestions of clinicians from the beginning of her pregnancy in the hope of being ‘guaranteed’ a healthy pregnancy outcome’. She conveyed her feelings in the statement below:

‘I had no control, yes, I had no control because all my faith, I had given it to the doctors.’ (Participant 7)

Similarly, another participant reported being in control at the beginning of her pregnancy:

‘I’m a planner. You know, I planned this whole experience, what it’s gonna be, all of that stuff.’ (Participant 6)

However, when her pregnancy became high-risk during labour, she felt powerless, reporting that her body had failed her, compelling her to release control to the clinicians:

‘I also released some control to them [*clinicians*], because I didn’t fight, I couldn’t really fight, I was too scared to, yeah.’ (Participant 6)

### Theme 3: Fear

Under the main theme, the following sub-themes emerged, ‘clinicians’ focus on the pregnancy risks as fear-eliciting’, ‘fear of losing the baby’ and ‘fear of losing one’s own life’. ‘Fear is a normal reaction to a real or imagined threat, which is considered to be an integral and adaptive aspect of development’ (Gullone 2000:429). All nine participants experienced some kind of fear during their pregnancies. Fear was therefore a central category that united all themes in this study (Table 2). It manifested itself in different ways relating to different aspects (‘clinicians’ focus on the pregnancy risks as fear-eliciting’, ‘fear of losing the baby’ and ‘fear of losing one’s own life’).

**TABLE 2:** Themes and sub-themes.

Themes	Sub-themes
1. Being labelled as ‘high- risk’	-
2. Locus of control	2.1. Mothers taking full control of birth outcomes: Defying biomedical rules 2.2. Loss of control: Releasing control to clinicians
3. Fear	3.1. Clinicians’ focus on the pregnancy risks as fear-eliciting 3.2. Fear of losing the baby 3.3. Fear of losing one’s own life
4. Hospitalisation	4.1. Sudden hospital admission 4.2. Prolonged waiting period for hospital admission 4.3. Lack of compassion from medical staff 4.4. Lack of support from medical staff 4.5. Support from medical staff: Showing compassion

Note: This Table presents these themes and subthemes, which are further elaborated in the article.

#### Sub-theme 3.1: Clinicians’ focus on the pregnancy risks as fear-eliciting

In the current study, ‘clinicians’ focus on the pregnancy risks as fear-eliciting’ refers to the tendency of practitioners to focus on worst-case scenarios when sharing information regarding participants’ diagnoses. This was frightening for the participants rather than instructive as shown in the excerpts below:

‘I was scared, I was traumatised because they told me, especially during that time of preeclampsia. They told me that it’s 50/50 chance that the baby will not make it, so I was very scared.’ (Participant 4)

‘What was on my mind was what he told me that if I happen to bleed, there’s a 50/50 chance that I would not live. That frightened me.’ (Participant 8)

For some participants, hearing the mention of ‘high-risk pregnancy’ among clinicians increased the degree of concern:

‘…that made me scared, when I kept hearing ‘high-risk’ ‘high-risk’, everywhere. It made me realise that I was in trouble. No, it was very difficult.’ (Participant 2)

#### Sub-theme 3.2: Fear of losing the baby

‘Fear of losing the baby’ was a theme that filtered through all interviews. This theme describes the degree of concern that participants had regarding the wellbeing of their babies. Participants addressed concerns regarding the survival of their pregnancies. Statements for this theme were categorised as ‘fear of losing the baby’ and ‘fear of the baby being harmed’. Most participants feared that something devastating could happen to their babies during labour. The following excerpts show how babies’ lives took precedence over that of participants:

‘Already you thinking about the child, is she still fine, you know, it was an emergency caesar, and they were saying, the child, so in my mind it was the child, I did not have time to concentrate on me mos.’ (Participant 4)

‘I thought I was going to lose him. The moment he was going to theatre, I was like wow, I’ve been there, I know how it feels. So, I thought that I was going to lose him because he was young and he had a small body. So yes, I thought I was going to lose him.’ (Participant 1)

‘You know they put the belt on, that you had to keep the baby monitoring, and sometimes that thing will beat so funny and I will scream, you know, that there’s something wrong with my child, you know. I, I was terrified that the umbilical cord could get stuck around his neck … because I’ve been through so much.’ (Participant 5)

In addition to fearing for their baby’s lives, participants also feared that their babies may be harmed by the physical pain that they were experiencing because of the pregnancy complications. Below are a few of these statements:

‘It’s the pain that I had that made me scared. When you have pain, you worry about what is happening to the baby, are they being harmed in any way. Are they also in pain? So, that made me scared.’ (Participant 9)

‘I was frightened about the fact that I was pregnant, at the same time, I’m having pain. What if that did something to the baby … so then that frightened me.’ (Participant 2)

#### Sub-theme 3.3: Fear of losing one’s own life

‘Fear of losing one’s own life’ was another sub-theme that emerged under the main theme of fear. This described the degree of concern that participants experienced about the possibility of their own death because of the pregnancy complications. Dying during a caesarean section was a common kind of fear that was related to the possibility of participants losing their own lives. This is expressed in the statements below:

‘I thought, I was going to die, I prayed a lot, I thought I was going to die, I don’t wanna lie, because to go to C-section.’ (Participant 1)

‘So, I was scared that I’m pregnant and, at the same time, they will perform an operation, what if I die? Yes, I was scared and frightened.’ (Participant 2)

On the other hand, there were those participants who expressed different kinds of fears that related to losing their own lives. Below is the statement of one such participant:

‘I was scared I was going to die because they told me that I had a shortage of blood, so after giving birth, they had to look for my blood type to give to me.’ (Participant 9)

### Theme 4: Hospitalisation

The theme ‘hospitalisation’ yielded five sub-themes which included ‘sudden hospital admission’, ‘prolonged waiting period for hospital admission’, ‘lack of compassion from medical staff’ and ‘support from medical staff: Showing compassion’.

#### Sub-theme 4.1: Sudden hospital admission

The mentioned theme refers to the participants’ ‘sudden hospital admission’, highlighting the unpredictable nature of a high-risk pregnancy and how pregnancy has come to be institutionalised. Most participants in the study reported that they were admitted during their routine antenatal care:

‘I woke up from my house, went to the hospital for a check-up [*antenatal visit*] and, all of a sudden, I’m being admitted.’ (Participant 8)

‘I think I was roughly just going for 6 months, and uhm, I went to work normal in the morning and my left eye started tearing … and my friend said to me, hey, your face is twisting and immediately my manager said to her, take her to the hospital.’ (Participant 5)

‘I think I was plus-minus 20 weeks, so I was told that my sugar levels were a little bit higher, so it was a local clinic that I was attending, so I was told that I will be transferred to a hospital. So, I went with the ambulance to the Mowbray hospital.’ (Participant 4)

When the severity of maternal complications increased, women were hospitalised for increased medical surveillance and intervention (Majella et al. 2019), and this was the case with participants in this study.

#### Sub-theme 4.2: Prolonged waiting period for hospital admission

The ‘prolonged waiting period for hospital admission’ described the extensive waiting time that participants experienced before being admitted. Participants frequently found it strange that they had to wait so long, especially considering that they had referral letters from prior medical facilities authorising their admission:

‘I was there very early in the morning, about past seven and I was told that I will be admitted, and I was kept there in the waiting room for like, for the whole day, I was only taken to the ward, around past seven in the evening.’ (Participant 1)

‘I thought that everything would be sorted out for me because I had an appointment letter from where I was attending my antenatal. So, I thought I would get there and be directed to my bed, but then instead I had to go through that whole process of taking a folder and getting my high-blood pressure checked. So, I got there 7 o’clock in the morning, and so had to wait the whole day.’ (Participant 7)

#### Sub-theme 4.3: Lack of compassion from medical staff

This theme highlighted the disregard shown by some clinicians towards the participants in this study. These practitioners showed little understanding of the challenging circumstances that the individuals were in. Although being yelled at or reprimanded by healthcare professionals is the most frequently cited form of hostility in research (Chadwick 2017), the current investigation revealed additional instances of abuse. For example, one participant was asked to clean up her own vomit in the midst of severe pain, as recounted below:

‘I was vomiting mos, so that nurse said, and I remember it was a Coloured [*mixed race*] lady. So, she told me to clean up my vomit, so I did that, I cleaned my vomit up and sorted everything out.’ (Participant 8)

Some participants such as Participant 7 reported being shouted at or scolded by a healthcare provider:

‘I was in too much pain, there’s that belt, I took that belt off. So, now it [*machine display*] had a straight line. When they came in the morning, yoh! that cherry [*nurse*] was shouting, “You took this thing out a long time ago!” She shouted and shouted.’ (Participant 7)

Other times, as this participant’s remark below demonstrated, individuals felt that their emotional needs were not being met:

‘It’s about the wound. They just check up, checked my pulse, my blood pressure, check my wound and give me tablets, that’s all. Nobody is worried about you, emotionally. Nobody’s asking about you emotionally; no, there’s no such there.’ (Participant 1)

#### Sub-theme 4.4: Lack of support from medical staff

This theme referred to the lack of compassion by practitioners demonstrated in not physically supporting women who were admitted to the hospitals and having difficulties in performing tasks that they would typically be able to accomplish on their own. The participants in the current research did not receive any help with making their beds or caring for their newborn babies following caesarean sections:

‘I had to take care of the baby, I had to make up my own bed. And I was like, if I was at home, I would have someone to assist me with the baby. Even with healing, it was going to be easy.’ (Participant 3)

‘Because the wound was still fresh, it was very painful. Nobody cared about you except for the doctors who came in and checked the wound and give you the tablets, of course, but everything else, you are on your own.’ (Participant 8)

A lack of compassion and support in government hospitals is a long-standing challenge in South Africa, which is unfortunately not unique to this study. The identification of a high-risk pregnancy already introduces additional stress and anxiety because of the uncertainty in a pregnant woman. It therefore becomes imperative for those diagnosed with risky pregnancies to receive psychological support, empathy and hope from clinicians (Janighorban et al. 2018).

#### Sub-theme 4.5: Support from medical staff: Showing compassion

Despite many participants experiencing indifference from clinicians, there were those who commented favourably and expressed appreciation towards healthcare providers. These participants acknowledged the emotional support provided by the medical staff.

For example, one participant painted a positive picture of the nursing staff who took care of her during her hospital stay. She described an environment where the door was always open for her to seek advice and to ask questions about her diagnosis whenever she felt anxious:

‘So, every time I had a problem I would connect with the nurses and was reassured.’ (Participant 9)

Similarly, Participant 6 reported that the medical team was friendly and welcoming. She described her experience as follows:

‘Everybody was nice, the doctors, the nurses. Everybody around me was nice.’ (Participant 6)

In addition to being friendly, Participant 4 reported that the hospital staff went as far as offering her a birthing partner as her husband was working in Limpopo during the time of the birth of her daughter:

‘I can say the staff was very, very nice … I didn’t have anyone, they offered someone during the process. The staff were very, very supportive.’ (Participant 4)

## Discussion

The study examined how women from resource-constrained areas experienced their pregnancy as well as interactions with the healthcare system. The four main themes that emerged included: (1) being labelled as ‘high-risk’, (2) locus of control, (3) fear and (4) hospitalisation.

Participants had varying perceptions and responses to the understandings of risk as well as the acceptance of surveillance that accompanied the risk label. Risk perception depended on two factors: a statistical evaluation of how probable an event will occur and a psychological component, which included how women felt about the risk (Rajbanshi, Norhayati & Nik Hazlina 2021). However, the study found that pregnant women are being classified as ‘high-risk’ entirely on statistical analysis, disregarding other elements including life experience and coping mechanisms (Rajbanshi et al. 2021).

Not all participants, however, were completely ‘compliant’ with the risk recommendations. Some participants asserted themselves against clinicians and defied the biomedical way of viewing pregnancy and the associated risks. These participants believed that healthcare professionals placed more emphasis on the chances of adverse outcomes of pregnancy such as death. As a result, when making decisions about their own welfare and the wellbeing of their children, they put less weight on the medical facts that were offered to them on the dangers and relied more on their own intuitive understanding.

Similar results were obtained by Lee et al. (2019) who also noted that mothers’ decisions were based on their personal assessments of the degree to which medical treatment was advantageous to their pregnancy and delivery. Thus, this does not imply that participants fully rejected medical intervention, rather they objected to interventions that they deemed unnecessary and which, in their opinion, would have inflated the risk (Hammer & Burton-Jeangros 2013).

However, it can be argued that the tendency of clinicians to emphasise the negative aspect of pregnancy is often rooted in the best of intentions as they may be preparing mothers for the worst-case scenario (Behruzi et al. 2014). The challenge with this view, however, is that once pregnancy is designated as ‘high-risk’, it compels clinicians to devote all the attention to medications and tests, neglecting the woman herself (Rodrigues et al. 2016).

Fear and anxiety emerged as dominant themes that were voiced relatively often by all participants. Nearly all participants used language that portrayed significant degrees of suffering. Most of these worries were related to self- and baby-related health concerns. Two common worries mentioned in the present study were fear of dying during a caesarean section or losing the baby because of pregnancy complications.

Like the current study, most studies that explored the emotional states of women diagnosed with a high-risk pregnancy found that fear and anxiety predominate (Yilmaz & Oskay 2021). It was also previously discovered that women living in resource-constrained environments are more vulnerable to anxiety which subsequently increases in pregnancy (Van Heyningen et al. 2017).

All participants in this study were subjected to sudden and unexpected hospitalisation, which was inevitable in the context of a high-risk diagnosis as previously reported by Sherfield (2021). Although the hospital environment is portrayed as a safe place for birth, the study revealed that public hospitals can be hazardous and risky for pregnant women in South Africa. For this reason, ‘It’s important to remember that exactly what is “offered” may be linked to the social positioning of women’ (Lowe 2016:129). Therefore, the study revealed that hospitalisation does not guarantee compassionate care for women from resource-constrained areas who are experiencing pregnancy complications.

The first barrier related to hospital admission was having to endure a 12-h waiting period before participants were taken into their wards, despite possessing referral letters. This is a persistent issue in South African public healthcare institutions that was highlighted in previous research (Maphumulo & Bhengu 2019; Young 2016); therefore, the current study is not the first to mention this.

Additionally, during hospitalisation, participants encountered hostile and cold healthcare professionals, which was evident in their lack of empathy and inability to physically aid the patients. Some of the accounts included being yelled at or reprimanded as well as not receiving help after a caesarean section, such as when making the bed. Similar narratives have been documented in other South African research (Chadwick 2017).

On the other hand, there were participants who described hospital medical staff as being kind and welcoming, painting a more favourable view of them. Although studies reporting satisfaction with public healthcare in LMICs are scant, a study conducted in Pakistan found some participants to be satisfied with the level of care that was provided (Manzoor et al. 2019). Similarly, a study conducted in Ethiopa reported that 41.2% of its participants were satisfied with the care that was provided to them (Kare, Gujo & Yote 2021). Moridi et al. (2020) observed that women required and highly valued the support and presence of healthcare professionals during the intrapartum period.

### Strengths and limitations

One of the key advantages of this study is the use of a qualitative research approach, which enabled the researcher to document the rich and in-depth experiences of mothers from resource-constrained communities who had their pregnancies classified as ‘high-risk’. Additionally, the study contributed to a body of knowledge on pregnant women’s interactions with the healthcare system. Moreover, the interviews provided mothers, many of whom were disclosing their experiences for the first time, with a therapeutic setting.

However, conducting interviews of such a sensitive nature virtually may be viewed as a limitation as factors, such as reflexivity, were difficult to ascertain during the data collection process because of the absence of body language. The flow of some interviews was interrupted by abrupt disconnections and interferences, which may have influenced the information shared. The study was limited to a small sample of women who resided in the Western Cape, in Cape Town’s resource-constrained communities. Hence, this may mean that different findings could emerge in other cultural contexts. Therefore, despite producing such rich data, findings cannot be generalised to all women experiencing high-risk pregnancies, as this is not the aim of a qualitative study.

### Recommendations

The government has made significant strides to increase the availability of healthcare services in South Africa. However, the delivery of these services still needs to be further investigated as they may not align with some of the needs of pregnant women. The identification of a high-risk pregnancy already introduces additional stress and anxiety because of the uncertainty in pregnant women. It therefore becomes imperative for those diagnosed with risky pregnancies to receive psychological support, empathy and hope from clinicians.

## Conclusion

The study has provided valuable insight into the challenging nature of high-risk pregnancies, which often leaves women feeling overwhelmed. It also revealed the different viewpoints that clinicians and expectant women have on treatment. Thus, the study showed that women’s unique needs and viewpoints are largely disregarded, highlighting the need for further research on the requirements of pregnant women, especially those with high-risk diagnoses. More crucially, allowing the women who participated in the research to share their stories has given them a voice.
